# Characterization of flavivirus infection in salivary gland cultures from male *Ixodes scapularis* ticks

**DOI:** 10.1371/journal.pntd.0008683

**Published:** 2020-10-05

**Authors:** Benjamin L. Kendall, Jeffrey M. Grabowski, Rebecca Rosenke, Mikayla Pulliam, Daniel R. Long, Dana P. Scott, Danielle K. Offerdahl, Marshall E. Bloom

**Affiliations:** 1 Biology of Vector-Borne Viruses Section, Laboratory of Virology, Rocky Mountain Laboratories, NIAID/NIH, Hamilton, MT, United States of America; 2 Rocky Mountain Veterinary Branch, Rocky Mountain Laboratories, NIAID/NIH, Hamilton, MT, United States of America; 3 Microscopy Unit, Research and Technologies Branch, Rocky Mountain Laboratories, NIAID/NIH, Hamilton, MT, United States of America; Baylor College of Medicine, UNITED STATES

## Abstract

Infected *Ixodes scapularis* (black-legged tick) transmit a host of serious pathogens via their bites, including *Borrelia burgdorferi*, *Babesia microti*, and tick-borne flaviviruses (TBFVs), such as Powassan virus (POWV). Although the role of female *I*. *scapularis* ticks in disease transmission is well characterized, the role of male ticks is poorly understood. Because the pathogens are delivered in tick saliva, we studied the capacity of male salivary glands (SGs) to support virus replication. *Ex vivo* cultures of SGs from unfed male *I*. *scapularis* were viable for more than a week and maintained the characteristic tissue architecture of lobular ducts and acini. When SG cultures were infected with the TBFVs Langat virus (LGTV) or POWV lineage II (deer tick virus), the production of infectious virus was demonstrated. Using a green fluorescent protein-tagged LGTV and confocal microscopy, we demonstrated LGTV infection within SG acinus types II and III. The presence of LGTV in the acini and lobular ducts of the cultures was also shown via immunohistochemistry. Furthermore, the identification by *in situ* hybridization of both positive and negative strand LGTV RNA confirmed that the virus was indeed replicating. Finally, transmission electron microscopy of infected SGs revealed virus particles packaged in vesicles or vacuoles adjacent to acinar lumina. These studies support the concept that SGs of male *I*. *scapularis* ticks support replication of TBFVs and may play a role in virus transmission, and further refine a useful model system for developing countermeasures against this important group of pathogens.

## Author summary

Powassan disease has greatly increased in frequency since its discovery in Powassan, Ontario in 1958. Powassan virus (lineage I; POWV) and Powassan virus lineage II (deer tick virus; DTV) are endemic to North America and there were 133 reported cases between 2009 and 2018, the majority since 2016. Nymphal and adult *Ixodes scapularis* ticks are thought to be the primary vectors of POWV/DTV to humans. However, little is known regarding DTV infection of male *Ixodes* ticks or their potential as vectors. In this study we characterized LGTV, a model tick-borne flavivirus, and DTV infection and propagation in male *I*. *scapularis* salivary gland cultures using an *ex vivo* organ culture system. This work provides insight into potential flavivirus transmission by the male *I*. *scapularis* tick.

## Introduction

The dramatic increase in tick-borne infections worldwide, specifically in North America, Europe, and Asia [1–4] has revived interest in many aspects of the corresponding infectious agents. Ticks transmit a vast array of infectious agents, including bacteria such as various *Borrelia* species and *Anaplasma*, parasites like *Babesia microti*, and viruses in several families [5–7]. The agents are delivered to the host by the bite of a feeding tick and are transmitted along with the complex mixture of bioactive molecules that is tick saliva [6, 7]. Because the salivary glands act as the final organ barrier to pathogen transmission [8], an in-depth understanding of tick salivary glands and tick saliva will be of paramount importance to the field.

The role of female ixodid ticks in the transmission of tick-borne flaviviruses (TBFVs) is well established [9, 10]. They will feed to repletion, over the course of several days, on a vertebrate host. A nymphal or adult female *Ixodes scapularis* infected with Powassan virus (POWV) can transmit the virus to a vertebrate host within 15 minutes after the onset of feeding. Infected ticks can also rapidly transmit virus to nearby co-feeding ticks via non-viremic transmission [11–14]. Furthermore, viruses can be passed transstadially between tick instars as well as transovarially from adult females to eggs [15, 16]. This complex life cycle is notable because it suggests that a virus population can be maintained in nature without a requirement for an infected vertebrate host.

While it has been observed that male ticks can become infected with POWV [15], their role in TBFV transmission and biology is not well understood. Male ticks typically take short, intermittent blood meals for nutrition and hydration and may feed sequentially on multiple hosts [17]. Such abbreviated feeding times may be sufficient to pass virus, if the dynamics of virus transmission by male ticks mirrors that of the females. In addition, the copulation process also provides a mechanism for pathogens to be transmitted from a male to a female tick and subsequent infection of the eggs [18]. This is because the male must salivate on the spermatophore for proper transfer to the female’s genital pore, thus providing another avenue for direct transmission [19, 20]. This study aims to further knowledge concerning the role that male ticks play in virus transmission, specifically by looking first at infection of salivary glands.

Salivary glands (SGs) of ixodid ticks have a structure similar to a bunch of grapes, with arborizing lobular ducts that terminate in glandular acini [21]. Both male and female ticks have several types of acini. Type I acini are agranular and believed to be responsible for osmoregulation [22–24]. Types II and III acini are comprised of cells with a variety of granules which contain the pharmacopeia of bioactive molecules [25, 26] that constitute tick saliva [22, 27–29]. We recently demonstrated that *ex vivo* SG cultures derived from female *I*. *scapularis* ticks can support TBFV replication and that virus localizes to cells of granular acini [30]. Our findings suggest that virus particles are in the SGs of infected ticks, thus offering an explanation for the rapid transmission of virus when feeding commences.

In the current study, we cultured SGs from adult male *I*. *scapularis* ticks to begin characterizing the role that male ticks play in virus transmission. The flavivirus of choice was Langat virus (LGTV), a TBFV which can be used as a model for tick-borne encephalitis virus, POWV, DTV, and other TBFVs of tropical origin such as Kyasanur Forest disease virus [31–33]. Our study found that SG cultures from male ticks were metabolically active in culture and that the replication of the LGTV occurred in granular acini [34, 35]. Male SG cultures also released infectious LGTV and DTV following infection, supporting the concept that the male tick may contribute to virus transmission.

## Materials and methods

### Male I. scapularis tick SG dissection and viability assay

Adult male *I*. *scapularis* ticks were obtained from Oklahoma State University (OSU) Tick Rearing Facility. They were aseptically treated, rinsed, dissected, and cultured as previously described [30]. Viability of the cultures was assessed by an alamarBlue (rezasurin salt-based) assay that measures the presence of cellular reducing agents (FMNH2, FADH2, NADH, NADPH, and cytochromes) [30].

### Preparation of virus stocks and virus quantitation

The propagation and quantitation of LGTV TP21 has been described [30, 37]. DTV (Powassan lineage II; Spooner strain) was obtained from Gregory Ebel, Colorado State University and was propagated using an MOI of 0.005 in African green monkey kidney cells (Vero, ATCC CRL-1587D). LGTV^GFP^ plasmid construction and rescue of GFP-expressing LGTV was performed as before [30, 38]. All virus stocks were aliquoted and stored at -80°C. Quantification of infectious virus titer by immunofocus assay was performed in Vero cells as described [30, 37].

### Infection of SG cultures and cell cultures

Dissected SGs were placed in wells of a 96-well plate with 100μl complete L15C-300 medium [39] and either mock-infected with complete L15C-300 medium only or inoculated with 5 x 10^5^ focus-forming units (ffu) of LGTV in 100μl complete L15C-300 medium. SG cultures were incubated for 1 h at 34°C with no supplemental CO_2_. Viral inoculum was then removed, replaced with fresh medium and incubated as before. At specified times, SGs were transferred to a microtube containing 1ml 4% paraformaldehyde and were fixed for 24 h at 4°C.

Vero and embryonic tick (ISE6, provided by T. Kurtti and U. Munderloh, University of Minnesota) [40] cell cultures were grown in T-25 flasks in Dulbecco’s Modified Eagle Medium (DMEM) and complete L15C-300, respectively. Culture medium was removed and 7ml of inoculate was added to each flask. Cells were either mock-infected with medium or LGTV-infected at an MOI of 1 then incubated for 1 hr in appropriate conditions (Vero: 37°C with 5% CO_2_; ISE6: 34°C with no CO_2_). The cells were rinsed with PBS and incubated in fresh medium until the indicated timepoints. Vero cells were detached using 0.25% trypsin/EDTA (Gibco) and ISE6 cells were pipetted off the flask surface without trypsin. Cells were then centrifuged at 300 rcf, 4°C for 5 minutes. The cells were resuspended in 5ml 1x PBS and recentrifuged. Cell pellets were finally resuspended in 5ml 4% paraformaldehyde fixative for at least 24 h at 4°C prior to further processing.

### Quantitation of infectious virus release from infected male SG cultures

Infection of SG cultures was performed as described above, using initial inoculum concentrations of 5 x 10^4^, 5 x 10^5^, or 5 x 10^6^ ffu of LGTV or 5 x 10^5^ ffu of DTV. Supernatants were collected at 3 and 36 hours post infection (hpi) and at 36-hour intervals thereafter to 240 hpi. Virus titer was then quantified via immunofocus assay [30, 37]. Attempts to rinse SG cultures following virus infection resulted in considerable sample loss. Therefore, the following method was adopted to determine viral growth curves. Virus inocula containing LGTV or DTV were separately added to wells containing SG cultures and to control wells containing no cultures. At the specified times, supernatants were collected, and virus was quantified by immunofocus assay. The logarithmic values of the titers were calculated for both the cultures and the control wells, and the value of the control wells were subtracted from the corresponding value of the wells with SG cultures.

### Confocal imaging for expression of LGTV^GFP^ in cultured SGs

The SG cultures were either mock-infected or virus-infected with 5 x 10^5^ ffu LGTV^GFP^ per SG pair and incubated until the indicated timepoints, using 3 SG pairs per experimental group to confirm the consistency of image results. Whole organ cultures were fixed for at least 24h at 4° C and then mounted on glass slides [28, 30] using Prolong Gold Antifade mounting agent (ThermoFisher Scientific). The preparations were held for 24 h at RT prior to being imaged using an LSM710 confocal microscope (Zeiss). Images were taken at 1.0X digital zoom with a frame size of 2,048 X 2,048 pixels. Objective lenses used were Ec Plan-Neofluor 20X numerical aperture (N.S.) air objective or a Plan-Apochromat 63X N.A. oil objective.

### Histopathology of SG cultures and cell cultures

SG cultures were infected, harvested, and fixed as described above. SGs were incubated for 3, 168, and 216 hpi, then the fixed cultures were subsequently rinsed in 1x PBS and transferred into 70% ethanol. 4 pairs of SGs per experimental group were pooled prior to downstream processing. Samples were manually processed through dehydration with a graded series of ethanol, cleared in Propar (Anatech), and embedded in Ultraffin paraffin (Cancer Diagnostics). Samples were sectioned and stained with hematoxylin and eosin as described previously [30].

Vero and ISE6 cell cultures were infected, harvested, and fixed as described above. Cell cultures were incubated for 24, 48, and 72hpi, then the fixed cell preparations were rinsed in 1x PBS and covered in a thin layer of Histogel matrix (Thermo Scientific, Kalamazoo, MI) which was then allowed to solidify at 4°C. Samples were then placed in tissue cassettes and processed using a VIP-6 Tissue Tek tissue system (Sakura Finetek, USA). Cells were embedded in Ultraffin paraffin polymer (Cancer Diagnostics, Durham, NC) and sectioned at 5 μm.

### Immunohistochemistry (IHC) to detect LGTV E protein

Anti-LGTV virus immunoreactivity was detected using anti-LGTV envelope (E) mouse monoclonal antibody 11H12, generously provided by Dr. Connie Schmaljohn, USAMRIID. 11H12 detected viral E protein and was used at a 1:50 dilution, as previously described [30]. The secondary antibody was a ready-to-use Discovery OmniMap anti-mouse horseradish peroxidase conjugate (Roche). Immunohistochemical staining and imaging of SGs was performed as previously described [30].

### Strand-specific in situ hybridization (ISH) to detect LGTV RNA

SG cultures or cell culture pellets were processed in paraffin and sectioned as described above. Positive sense (genomic) strand or negative sense (complementary) strand viral RNA were detected by using the RNAScope 2.5 assay on the Discovery Ultra instrument utilizing RNAscope V-Langat and RNAscope V-Langat-sense probes (Advanced Cell Diagnostics, Newark, CA).

### Transmission electron microscopy

Mock-infected or LGTV-infected male SG cultures were collected at 3 hpi, 168 hpi, and 216 hpi. 4 SG pairs were collected per timepoint and infection type and were subsequently processed for study by transmission electron microscopy as previously described [28].

## Results

### SG culture viability

**Fig 1:**
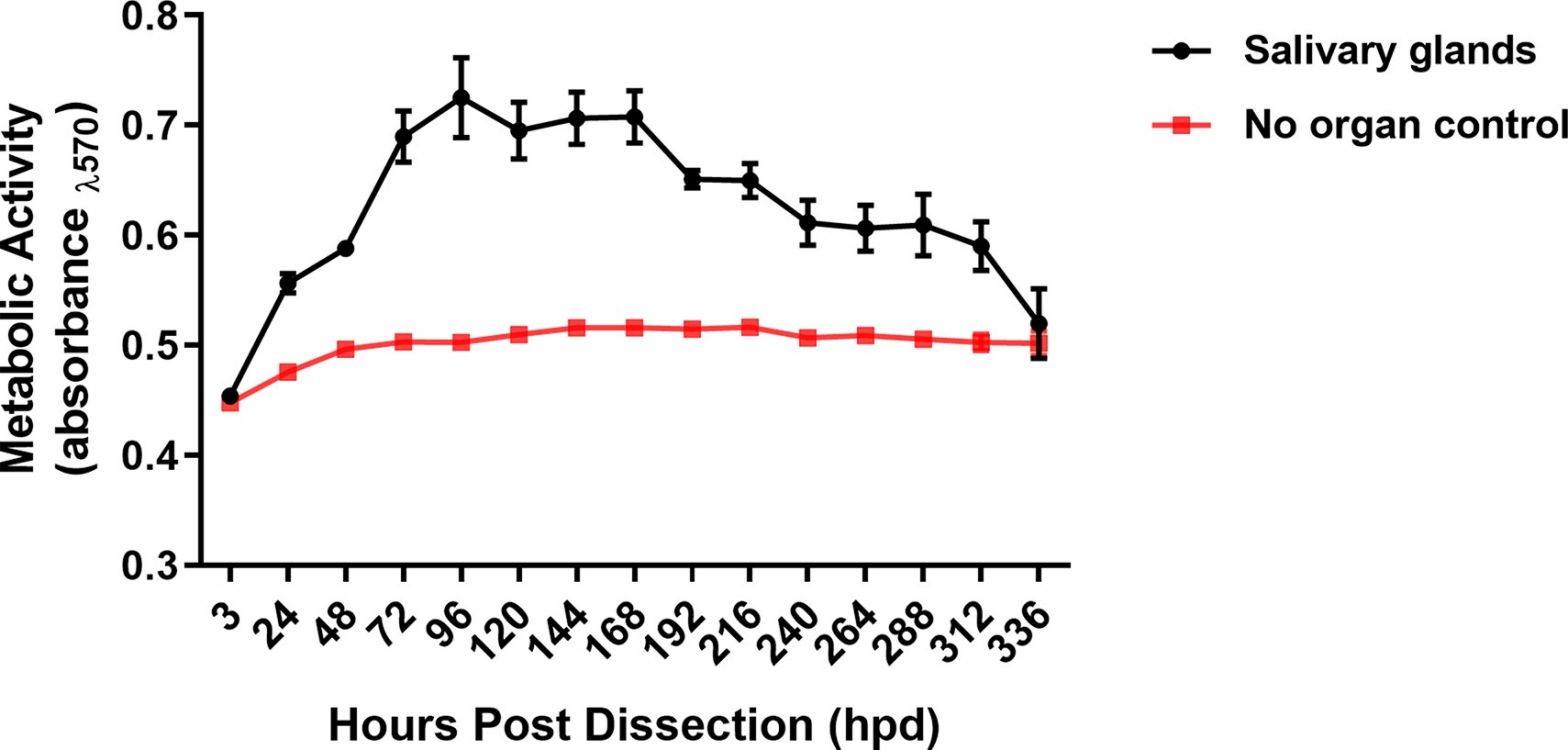
Metabolic activity of SG cultures from male ticks. Metabolic activity of male *Ixodes scapularis* salivary gland (SG) cultures was assessed using a fluorescent resazurin salt-based viability assay. Absorbance values were taken at 3 and 24 hours post-dissection (hpd), then every 24 hours thereafter to 336 hpd. Each experimental well contained 1 pair of SGs and was accompanied by a corresponding no-organ control well. The experiment was completed with 3–4 biological replicates per timepoint. Error bars indicate standard errors of the means, and data are representative of 3 machine replicates for each sample during viability reads.

Aseptic dissection of male *I*. *scapularis* SGs was performed and SG pairs were placed in complete L15C-300 media. The ability of male SG cultures to reduce resazurin salt as a measure of metabolic activity was then evaluated using a resazurin-based viability assay [30].Cultures of male *I*. *scapularis* SGs were metabolically active for nearly two weeks **(Fig 1)**, and activity peaked between 72 hours post dissection (hpd) and 168hpd, similar to the metabolic activity in female SG cultures [30]. These results confirmed that *ex vivo* male tick salivary gland cultures were a suitable model for subsequent studies, and we selected 216 hours in culture as an experimental end-point time.

### TBFV release from infected SG cultures

**Fig 2:**
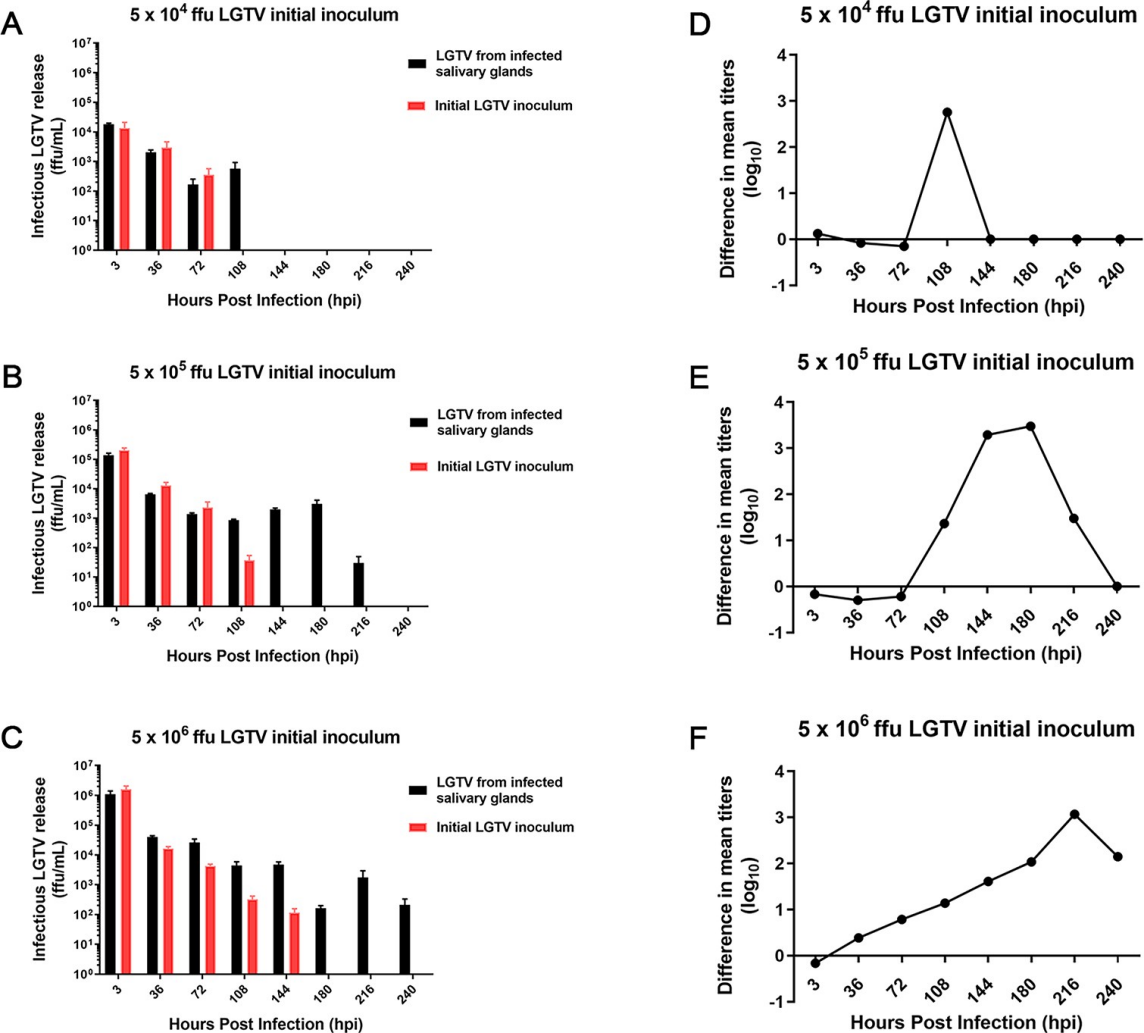
LGTV titer from infected male SG cultures. Infectious virus release from Langat virus (LGTV)-infected male *I*. *scapularis* salivary glands (SGs) was quantified using immunofocus assays. Experimental and control samples were inoculated with 5 x 10^4^ (A), 5 x 10^5^ (B), and 5 x 10^6^ (C) focus-forming units of LGTV. Immunofocus assays were performed using supernatants collected from experimental wells with SGs infected with LGTV and control wells with LGTV inoculum and no SGs. Error bars represent the standard error of the means. Viral titers per timepoint were averaged and the log_10_ values were calculated. Initial inoculum (control) logarithmic values were then subtracted from the corresponding infectious virus release logarithmic values per timepoint to visualize timepoints of peak virus release (D-F). These data are representative of 2–3 biological replicates and 2 technical replicates per timepoint. Data were processed using GraphPad Prism software v7.04.

**Fig 3:**
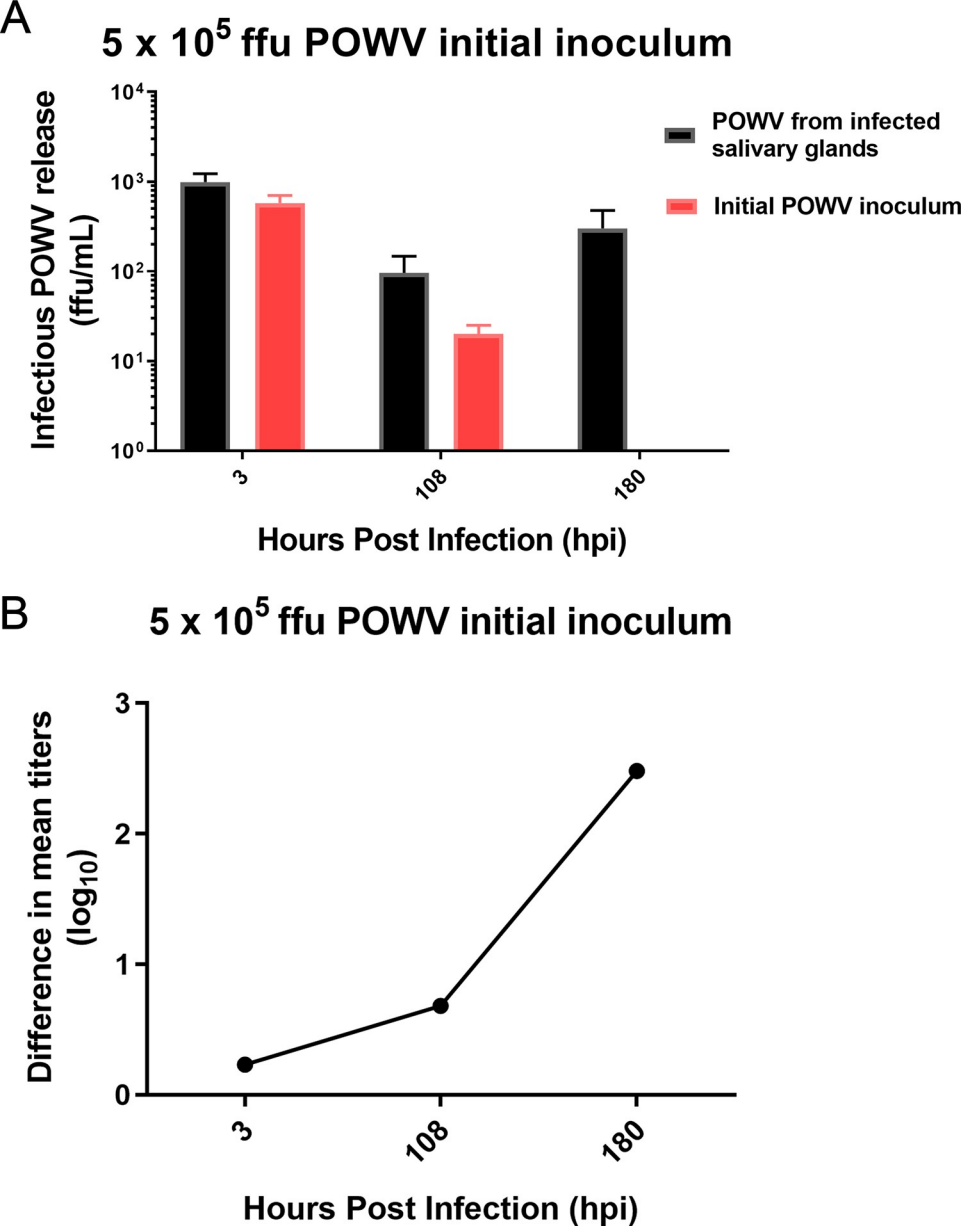
DTV titer from infected male SG cultures. Infectious virus release from Powassan virus lineage II (DTV)-infected male *I*. *scapularis* salivary gland (SG) cultures was quantified using immunofocus assays. Experimental and control samples were inoculated 5 x 10^5^ focus-forming units of DTV. Immunofocus assays were performed using supernatants collected from experimental wells with SGs infected with DTV and control wells with DTV inoculum and no SGs (A). Error bars represent the standard error of the means. Viral titers per timepoint were averaged and the log_10_ values were calculated. Initial inoculum (control) logarithmic values were then subtracted from the corresponding infectious virus release logarithmic values per timepoint to visualize timepoints of peak virus release (B). Data is representative of 3–4 biological replicates and 2 technical replicates per timepoint. Data were processed using GraphPad Prism software v7.04.

After dissection, male SGs were blotted onto 3x3mm sections of adsorbable gelfoam (Pfizer) and infected with either LGTV or DTV and incubated and then harvested at 3hpi, and 36-240hpi at 36-hour intervals. Control wells contained gelfoam and virus with no organs present. Male SG cultures infected with LGTV produced infectious virus. Plotting the difference in logarithmic values of the experimental and control groups indicated peak virus titer of roughly 1000 ffu/ml between 108 and 216 hpi at various inoculum concentrations **(Fig 2).** We also infected male tick SG cultures with 5 x 10^5^ ffu DTV and again observed release of infectious virus at 180hpi **(Fig 3)**. Thus, the male SG cultures were permissive for both LGTV and DTV.

### Identification of tissue types susceptible to LGTV infection

**Fig 4:**
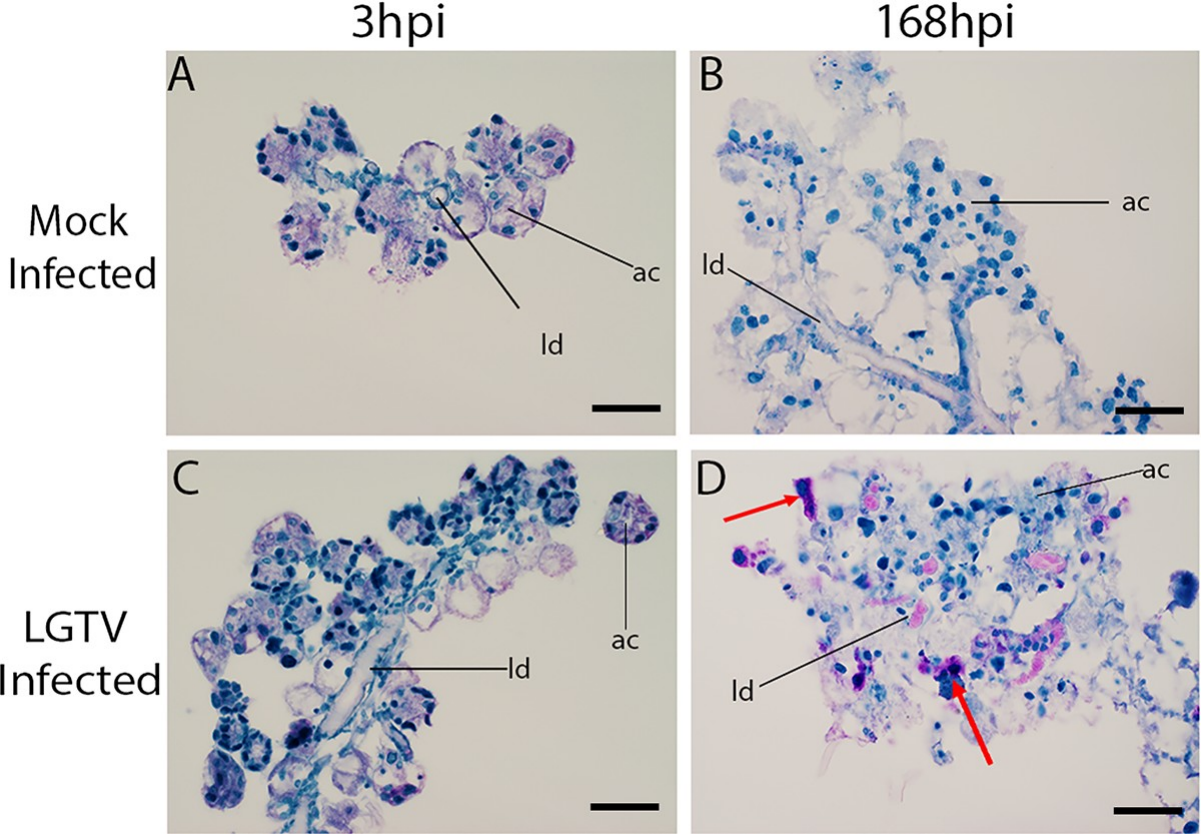
Immunohistochemical staining of LGTV-infected SG cultures from male ticks. We stained mock- and Langat virus (LGTV)-infected salivary glands (SGs) (infected at 5 x 10^5^ focus-forming units) for detection of LGTV envelope (E) protein in LGTV-infected SG cultures from male *Ixodes scapularis* ticks. Images were taken at 40X magnification and display a single SG from a pool of 4 SG pairs per experimental group. Each SG measured approximately 1mm in length, and each granular acinus measured roughly 40–50 microns in diameter. Red arrows indicate E protein expression as denoted by deep purple coloring. ld–lobular duct, ac–acinus. Mock-infected samples were treated identically to LGTV-infected samples to allow for identification of potential nonspecific staining. Scale bars are representative of 40 microns.

Both male and female *I*. *scapularis* SGs are composed primarily of salivary ducts and 3 types of acini. Cells in acinus types II and III contain granules in which the active components of saliva are found. We employed IHC for LGTV E protein to identify the specific site of virus infection. E protein was found in cells of granular acini as well as cells lining the lobular duct at 168hpi **(Fig 4)**.

**Fig 5:**
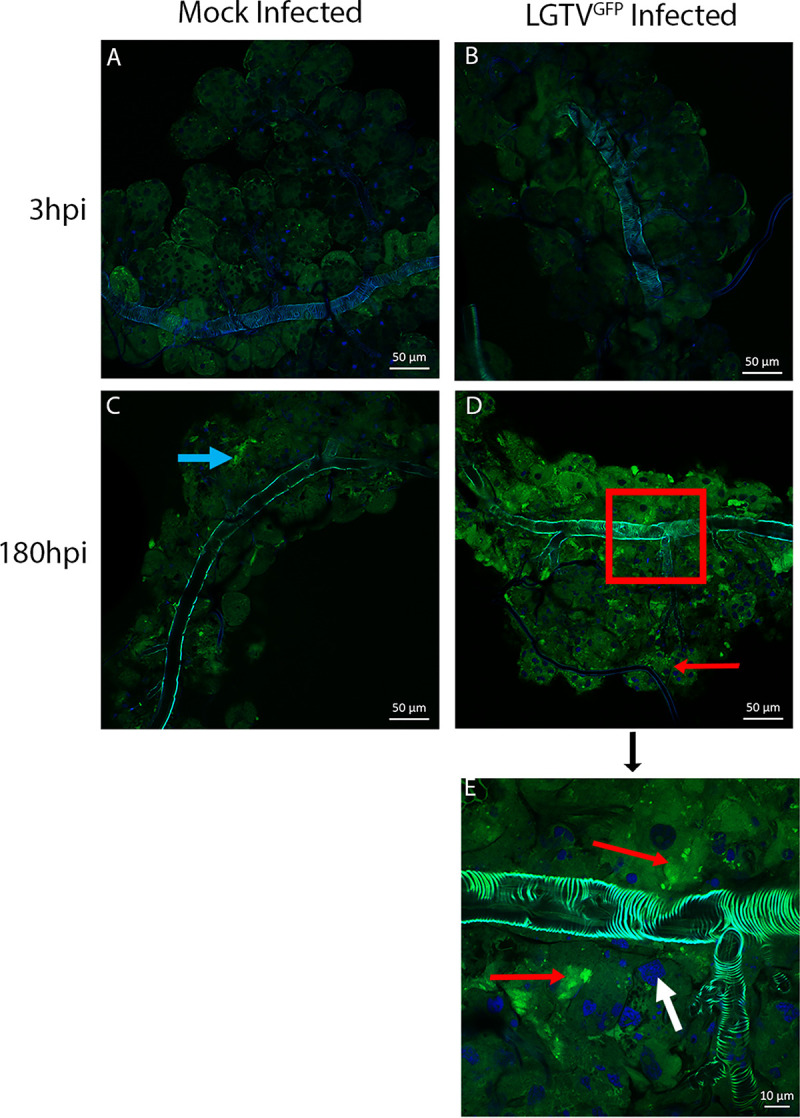
Localization of LGTV^GFP^ in infected SG cultures from male ticks. Whole mounts of mock- and green fluorescent protein tagged-Langat virus (LGTV^GFP^)-infected salivary glands (SGs) from male *Ixodes scapularis* ticks were imaged via confocal microscopy. Each image displays a single SG from a pool of 3 SG pairs per experimental group. A-D images were taken at 40X and insert E was taken at 63X magnification, representing the portion of image D outlined with the red box. Image processing was consistent among respective timepoints. LGTV^GFP^-infected SGs shows both discrete puncta as well as broad aggregations of GFP expression in granular acini. Red arrows indicate LGTV^GFP^, blue arrows indicate autofluorescence, and white arrows indicate DAPI stained cell nuclei. Samples from mock-infected organs were shown in order to compare SG autofluorescence.

Similar results were observed by confocal microscopy of SG cultures infected with green fluorescent protein-tagged LGTV (LGTV^GFP^) **(Fig 5)**. Mock-infected SG cultures were used as a comparison to control for autofluorescence. We observed specific GFP signal in cells lining the lobular duct as well as in acini located distally relative to the main lobular duct. Based on general location and morphology of the E protein-positive acini as well as the location of LGTV^GFP^ expression in the SGs, it seemed very likely that LGTV was infecting cells in both types II and III acini.

### Localization of viral RNA

**Fig 6:**
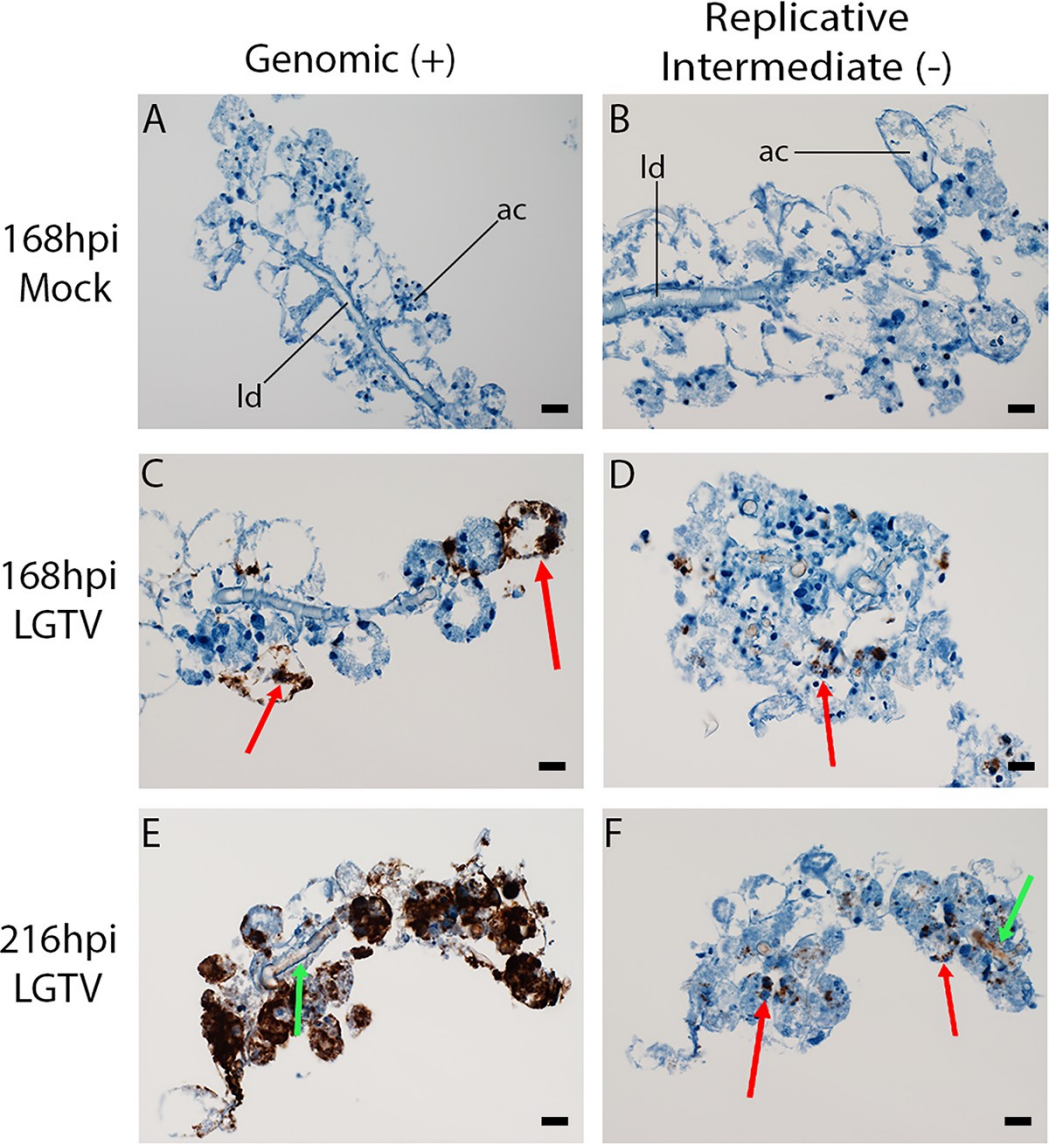
Strand-specific *in situ* hybridization of LGTV RNA in infected SG cultures from male ticks. Male *Ixodes scapularis* salivary gland (SG) cultures were infected with 5 x 10^5^ focus-forming units of Langat virus (LGTV) and stained with RNAscope V-Langat and RNAscope V-Langat-sense probes (Advanced Cell Diagnostics, Newark, CA) to reveal the location of viral RNA. Images were taken at 40X magnification and display a single SG from a pool of 4 SG pairs per experimental group. Each SG measured approximately 1mm in length, and each granular acinus measured roughly 40–50 microns in diameter. Red arrows indicate localizations of LGTV RNA in acini and green arrows indicate LGTV RNA on cells lining the lobular duct. ld–lobular duct, ac–acinus. Sections in panels A, C, and E were treated with an “antisense” probe to reveal location of plus-sense strand LGTV RNA in brown. Sections in panels B, D, and F were treated with the “sense” probe to reveal location of minus-sense strand LGTV RNA, an obligatory marker for viral replication. Mock-infected samples at 168 hours post infection (hpi) and also 216 hpi (not shown) exhibited no reaction with either probe. Scale bars are representative of 40 microns.

An unequivocal marker for TBFV replication is the demonstration of genomic (+ sense strand) and replicative intermediate (- or complementary sense strand) RNA. For this reason, we performed strand-specific ISH on sections of LGTV-infected SG cultures. While there was no signal for either strand of LGTV RNA in mock-infected samples **(Fig 6A and 6B)**, at 168 hpi, a strong signal for genomic strand RNA was evident in some granular acini **(Fig 6C)**, and replicative intermediate strand could also be detected **(Fig 6D)**. At 216 hpi, most if not all granular acini were intensely positive for genomic LGTV RNA **(Fig 6E)**. Replicative intermediate RNA was present in similar locations as well as along the lobular ducts **(Fig 6F)**. These results confirmed that the LGTV genome was actively replicating in granular acini in the SG cultures, which marked the granular acini as sites of replication.

At the later time point, the ISH signal from the genomic sense strand was demonstrably stronger than that from the complementary strand, a finding also noted in LGTV-infected Vero and ISE6 cells **(S1 Fig)**. This reflects the presence of + sense genomic strand RNA in virions as well as in replicative intermediates and translation complexes while the - sense strand is present only in duplex replicative intermediates [41].

### Identification of LGTV particles in SG cultures

**Fig 7:**
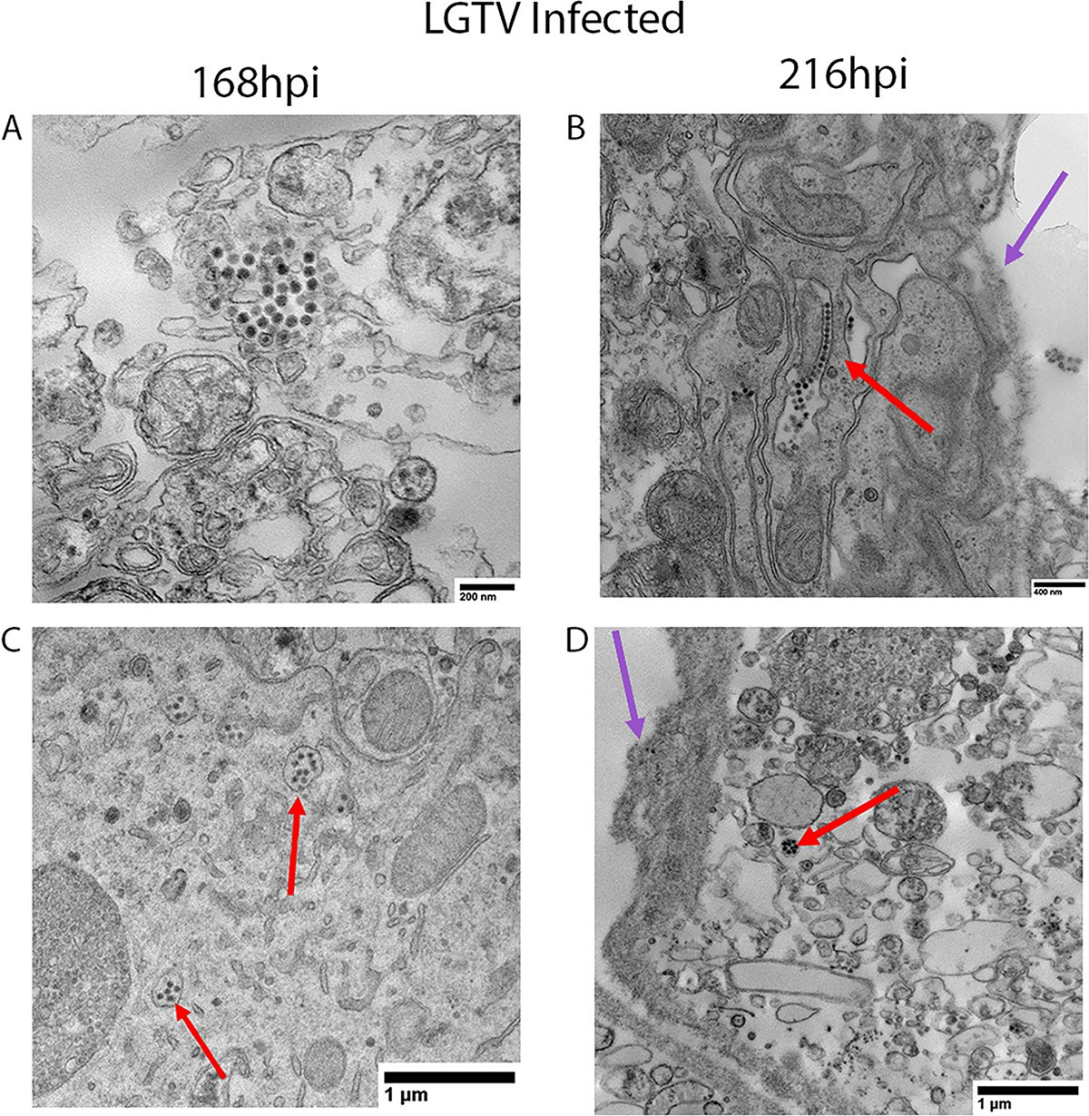
Transmission electron microscopy of LGTV-infected SG cultures from male ticks. Male *Ixodes scapularis* salivary gland (SG) culture samples were prepared for transmission electron microscopy at 168 hours post infection (hpi) and 216hpi. Each image displays a single SG from a pool of 3 SG pairs per experimental group. In LGTV-infected samples, virus particles were packaged in single-membrane-bound compartments (A, C, and D) or extracellular (B) and had a diameter of approximately 41-42nm. Red arrows indicate virus particles and purple arrows denote the lumen of the acinus. Panel B displays virus particles that appear to be extracellular.

Finally, we examined ultra-thin sections of LGTV-infected SG cultures by transmission electron microscopy (TEM) in order to visualize virus particles. Virions were readily observed adjacent to the lumen of granular acini (**Fig 7**) and in single-membrane-bound compartments (**Fig 7C and 7D**). We also observed extracellular virions **(Fig 7B)**. These particles were present at both 168hpi and 216hpi. Virions were approximately 41-42nm in diameter, consistent with the 40-50nm size of a flavivirus virion [42, 43]. The ultrastructure of the ER membranes of infected male SG cultures was similar to those of LGTV-infected ISE6 cultures, in that ER membranes appear largely unaffected compared to mammalian cells which exhibit substantial ER membrane expansion [37]. We concluded that LGTV virions are present in the SG cultures, further confirming virus infection and replication in *ex vivo* SG cultures **(Fig 2)**.

## Discussion

The increased number of existing tick-borne disease cases and emerging diseases vectored by *Ixodes scapularis* merits expanded research into exactly how those pathogens are transmitted [9]. Our study on TBFV infection of *ex vivo* salivary gland cultures from male ticks focused on one aspect of this topic–the role of male ticks in the transmission of virus. Infected ticks can infect mammalian hosts and other co-feeding ticks with TBFVs in a shorter interval than can be accounted for by *de novo* virus replication. This is possibly due to the ability of tick SGs to harbor virus that can be quickly released into the saliva. Furthermore, as we showed previously, *ex vivo* SG cultures provide a highly controlled model for studying infection and related processes [28, 30, 36].

The demonstration that infectious virus was generated in cultures from male *I*. *scapularis* SGs infected *in vitro* mirrors our results from female ticks [28,30]. While it has been previously established that live male ticks can become infected by POWV [15], no study has shown LGTV or DTV transmission from male ticks. We observed that infected cultures from male SGs could produce infectious LGTV and DTV (**Figs 2 and 3**), thus providing a potential mechanism for the rapid appearance of virus in saliva. Along with TBFV infection, SG cultures were mock-infected with a medium-only control. Future studies can incorporate a control involving noninfectious flavivirus particles (e.g. UV-inactivated, irradiated, heat inactivated) [31, 44, 45] to study infection kinetics and the potential influence of defective interfering particles. Taken together, our results suggest that male *I*. *scapularis* ticks are likely to be viable vectors of TBFVs.

Types II and III SG acini secrete important components of saliva, and the identification of virus in these acini suggests an important mechanism for TBFV transmission. Confocal imaging of LGTV^GFP^-infected SG cultures localized virus to the granular acini, as previously noted in cultures from female ticks [28, 30]. Small puncta of GFP signal were also visible in cells lining the lobular duct, but acinus types II and III contained the most substantial GFP signal (**Fig 5D and 5E**). IHC for viral E protein yielded similar findings, with E protein being primarily located in granular acini and to a lesser extent in cells lining the lobular ducts **(Fig 4D)**. Results of staining for the E protein do not exclude the possibility that cells are also taking up noninfectious virus. Therefore, staining for a nonstructural protein (such as LGTV NS5) may allow for identification of only infectious virus aggregates in the SG cultures. We could not exclude that agranular type I acini also became infected, but our primary focus is on the granular acini types II and III. The results closely resemble those we obtained for female *I*. *scapularis* SGs [28, 30].

ISH analysis of infected SG cultures showed both genomic and replicative intermediate viral RNA in both the acini and portions of the lobular duct. The demonstration of duplex replicative form RNA in granular acini marks them as sites of actual replication [42, 46]. We estimated that genomic strand was present at a 10 to 100-fold higher amount than the replicative intermediate strand LGTV RNA **(Figs 6 and S1)**. This was expected because the genomic strand exists not only in duplex replicative form but is also present in virions and translation complexes [46–48]. Taken together, all these results strongly suggest that active TBFV replication occurs in male SGs [49, 50].

Male ticks take substantially shorter blood meals than females; however, our results support the idea that male *I*. *scapularis* may still be able to transmit TBFVs. Moreover, we were able to identify the cellular localization of virus particles in LGTV-infected male SGs using TEM. Some of the particles were extracellular and others were enclosed in a single-membrane-bound compartment, perhaps for intracellular transport as occurs in the typical flavivirus life cycle (**Fig 7B)** [41, 42, 51]. These findings also parallel what we observed in infected SG cultures from female *I*. *scapularis* [28].

Further study of TBFV infection in male *Ixodes* ticks is warranted to better define their role in the biology of these pathogens. For instance, single-cell transcriptomic analysis of infected SG cultures might help identify potential cellular determinants of viral replication, and perhaps even specific target cell types within the acini. Nonetheless, isolating single viable cells from tick organs, such as those from salivary glands, has been shown to be a tedious process and would most likely require an high degree of optimization [52]. This analysis, along with proteomic profiling, would help contribute to understanding functionality of *ex vivo* SG cultures versus *in vivo* SGs at the molecular level. Additionally, SGs from males of certain species of ticks have a fourth type of acinus [53, 54], and the sexual exclusivity of this acinus type suggests a role in sexual reproduction. It is known that extra saliva is required for the transfer of the male tick’s spermatophore to the female [19, 20]; if type IV acini fulfill this role and can be infected by TBFVs, this might provide another route of pathogen transmission. Obviously, work like this would be followed by *in vivo* studies to confirm our suggestion that male *I*. *scapularis* ticks can transmit TBFVs to vertebrate hosts and potentially to female ticks during copulation.

Future studies with the *ex vivo* SG infection model can be performed to determine how well it represents infection kinetics within whole tick *in vivo*. Even studies comparing excised *ex vivo* SG infection versus other *ex vivo* setups, such as the backless tick method, may provide similar or different infection phenotypes [55]. The described *ex vivo* cultures here allow identification of acinus types that are susceptible to virus infection; however, the intact physical barriers and cellular responses (e.g. metabolism, immune) of a whole tick may potentially affect virus dissemination. Studies comparing barriers to SG infection *ex vivo* versus *in vivo* would be insightful. These can include an analysis of the genome copy ratios over time in SG cultures which would establish a more comprehensive view of virus replication *ex vivo*. Additional variables such as viruses overcoming the midgut barrier, the rate of transstadial transmission between nymph and adult male *I*. *scapularis*, viral interaction with humoral and cellular immune system components of the tick hemolymph and SGs, and additional types of organ viability assays can provide numerous directions to further develop this model. Furthermore, this *ex vivo* organ model can be expanded into other tick species, including vectors of important human and livestock pathogens in tropical regions of the world.

In conclusion, we have successfully established an *ex vivo* organ culture system for adult male *I*. *scapularis* SGs and identified SGs as target organs for LGTV and DTV replication. We also identified the granular acini of the SG as specific sites for LGTV infection and suggested that male *I*. *scapularis* ticks may be able to transmit TBFVs. This study provides a novel analytic tool for examining the role of the male ticks in pathogen transmission.

## Supporting information

S1 Fig *In situ* hybridization of LGTV RNA in infected cell cultures. To validate ISH results **(Fig 6),** we infected cultures of the *Ixodes scapularis* embryo-derived cell line ISE6 with Langat virus (LGTV) and treated them with RNAscope V-Langat and RNAscope V-Langat-sense probes (Advanced Cell Diagnostics, Newark, CA). All panels show cell cultures infected at an MOI of 1 and were incubated for 72 hours post infection. Samples A and C were treated with antisense probe to show (+) strand LGTV RNA in brown. Samples B and D were treated with sense probe to show presence of [19] (-) strand LGTV RNA in brown. Mock-infected samples (not shown) were treated in the same manner and did not show any non-specific signal. Scale bars are representative of 120 microns.
(TIF)
