# Supplementary figures and images for: Characterization of flavivirus infection in salivary gland cultures from male Ixodes scapularis ticks
Source: PLoS Negl Trop Dis. 2020 Oct 5;14(10):e0008683. doi: 10.1371/journal.pntd.0008683 (PMC7561187; doi:10.1371/journal.pntd.0008683)

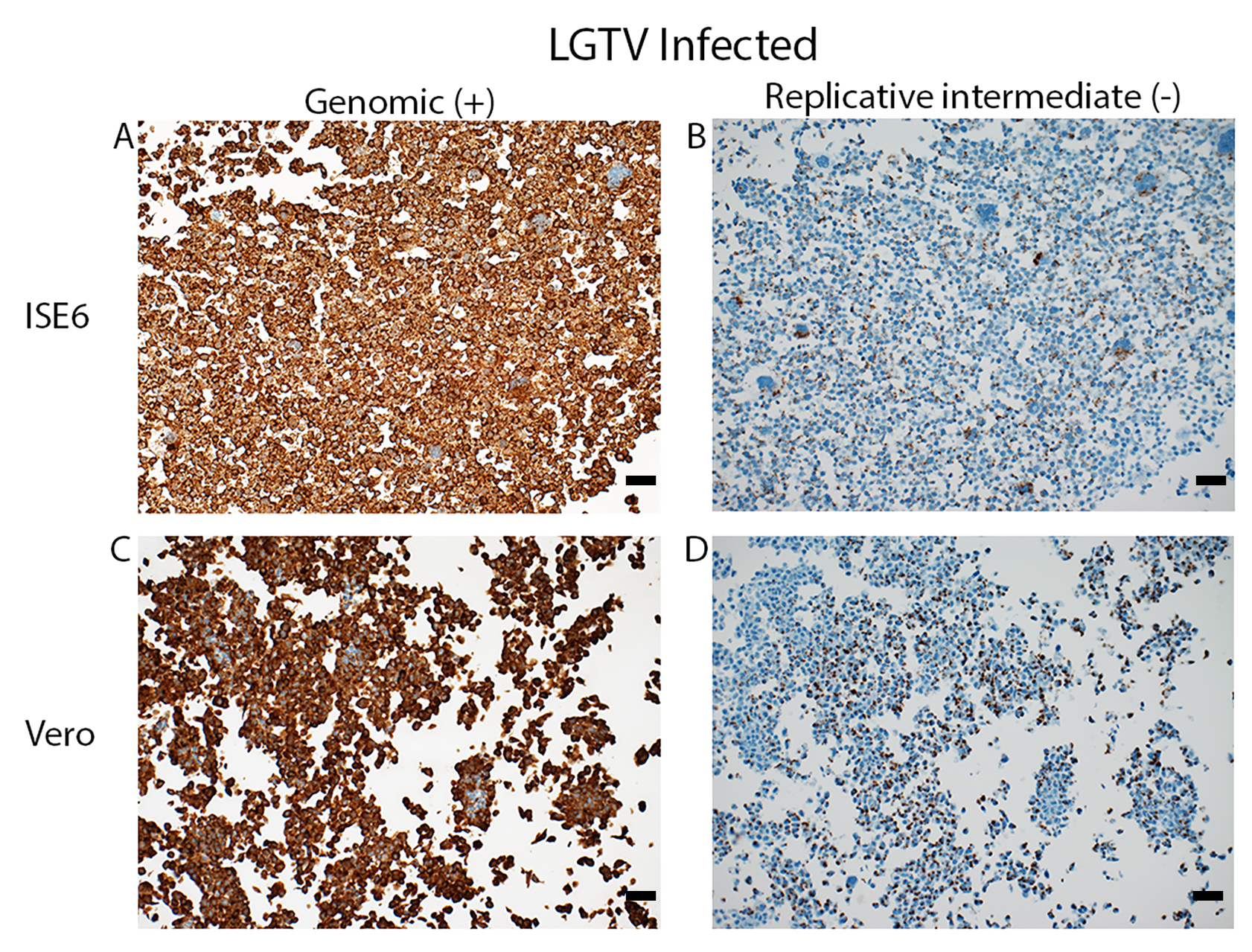

Supplement: S1 Fig — To validate ISH results (Fig 6), we infected cultures of the Ixodes scapularis embryo-derived cell line ISE6 with Langat virus (LGTV) and treated them with RNAscope V-Langat and RNAscope V-Langat-sense probes (Advanced Cell Diagnostics, Newark, CA). All panels show cell cultures infected at an MOI of 1 and were incubated for 72 hours post infection. Samples A and C were treated with antisense probe to show (+) strand LGTV RNA in brown. Samples B and D were treated with sense probe to show presence of [19] (-) strand LGTV RNA in brown. Mock-infected samples (not shown) were treated in the same manner and did not show any non-specific signal. Scale bars are representative of 120 microns. (TIF) [file pntd.0008683.s001.tif]
